# Treatment of urinary calculi after Yang-Monti ileal ureter reconstruction: a case report

**DOI:** 10.1186/s12894-019-0443-y

**Published:** 2019-01-30

**Authors:** Zhenxing Wang, Zhaolin Sun, Guangheng Luo, Ye Tian, Xiushu Yang

**Affiliations:** 1 Basic Medical College, Guizhou Medical University, Guiyang, 550004 China; 2 Department of Urology, Guizhou Provincial People’s Hospital, No. 83 East Zhongshan Road, Guiyang, 550002 Guizhou China

**Keywords:** Ureter, Ureteroscopy, Urinary calculi, Holmium, Lasers

## Abstract

**Background:**

The development of ureteral calculi after Yang-Monti ileal ureter reconstruction has not been reported. This study was performed to explore the safety and effectiveness of ureteroscopy combined with laser lithotripsy in the treatment of ipsilateral lower ureteral calculi and lower calyceal calculi after Yang-Monti ileal ureter reconstruction.

**Case presentation:**

A 48-year-old man was admitted to our hospital with ipsilateral distal ureteral calculi and ipsilateral lower calyceal calculi. One year prior to this admission, the patient had undergone Yang-Monti ileal ureter reconstruction due to long-segment ureteral stenosis. After conservative treatment failed, we used a rigid ureteroscope with a holmium laser to break up the distal ureteral calculi, and successfully removed the renal calculi with a digital flexible ureteroscope and basket extractor.

**Conclusion:**

The successful outcome of the present case suggests that ureteroscopy combined with laser lithotripsy is a valuable option for the management of urinary calculi following Yang-Monti ileal ureter reconstruction.

## Background

The development of ureteral calculi after Yang-Monti ileal ureter reconstruction has not been reported. The treatment of ureteral stones is technically challenging, especially for surgeons with minimal experience in such procedures. We used ureteroscopy combined with laser lithotripsy to successfully treat a patient with ipsilateral distal ureteral calculi and ipsilateral lower calyceal calculi after Yang-Monti ileal ureter reconstruction due to long-segment ureteral stenosis.

## Case presentation

**Fig. 1:**
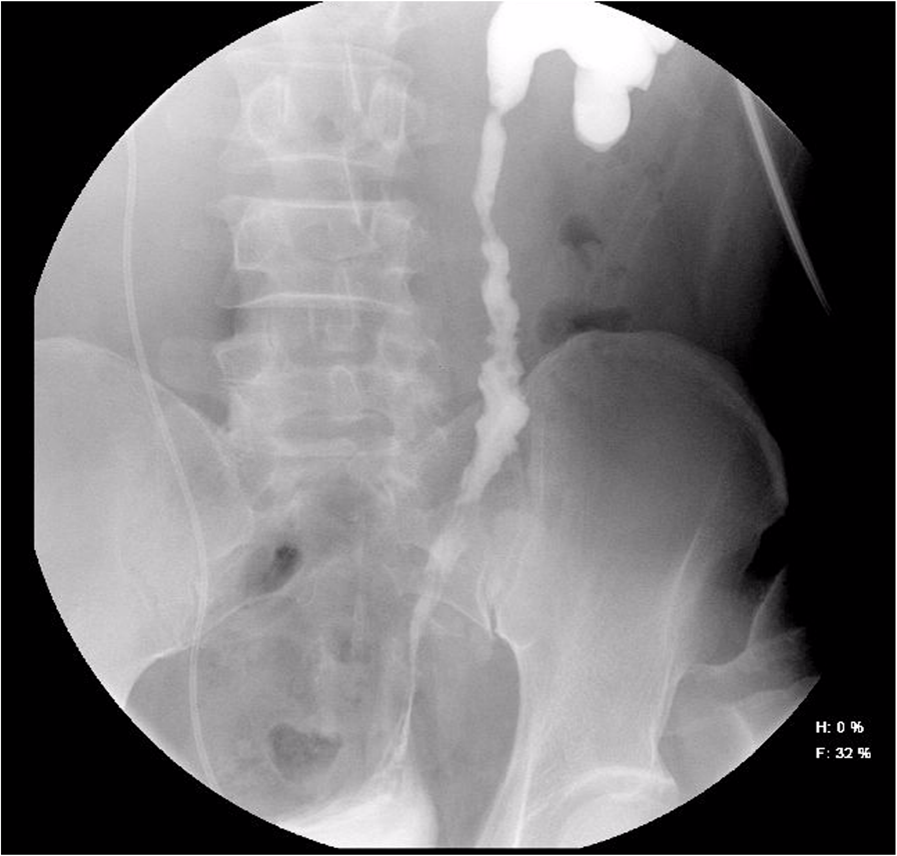
Left ureteral patency at 6 months after Yang-Monti ileal ureter reconstruction

The patient was a 48-year-old man with a 4-year history of ureteral calculi and a 2-year history of ureteral stenosis for which he had undergone multiple surgeries. Ureteral calculi recurred many times, resulting in repeated ureteral stricture that was treated three times by ureteroscopic holmium laser lithotripsy, and twice by ureteroscopic balloon dilation. He had been admitted to our hospital for left ureteral stenosis in June 2015. Examination at that time revealed a 20-cm stenosis in the middle and lower segments of the ureter. Noncontrast computed tomography showed inflammation and adhesion around the kidney as a result of multiple ureteral surgeries, making the patient unsuitable for autologous kidney transplantation. Yang-Monti ileal ureter reconstruction was performed, and the 6-month postoperative examination revealed left ureteral patency (Fig. 1), stable renal function, normal electrolyte levels, and no obvious mucus-like flocculation in the urine.

**Fig. 2:**
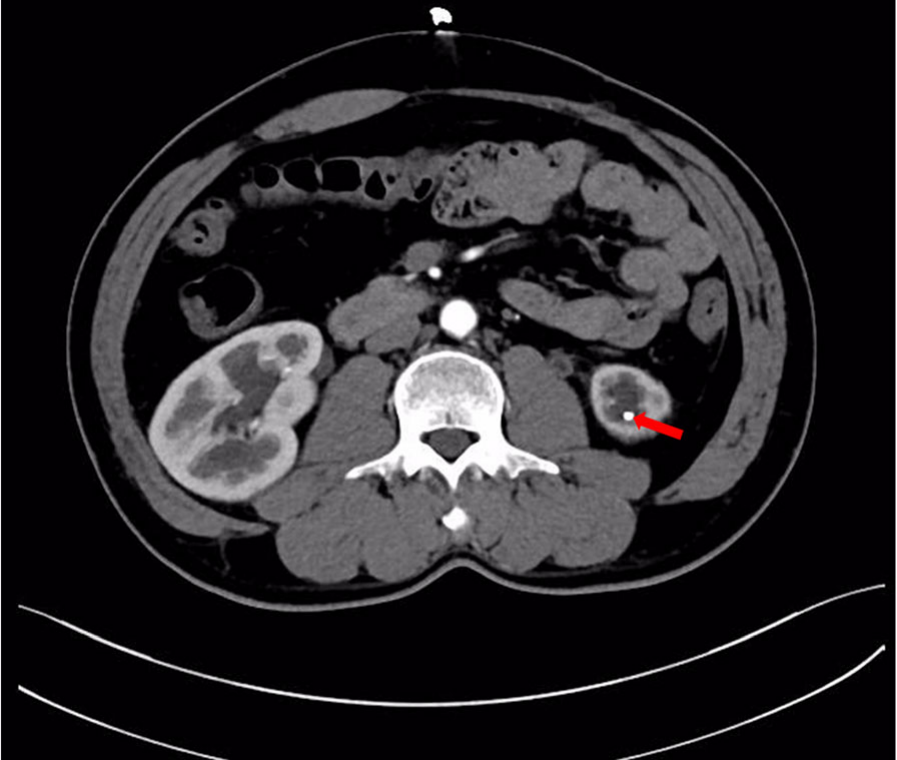
Two nodule-shaped high-density shadows in the lower renal calyx (indicated by the arrows)

**Fig. 3:**
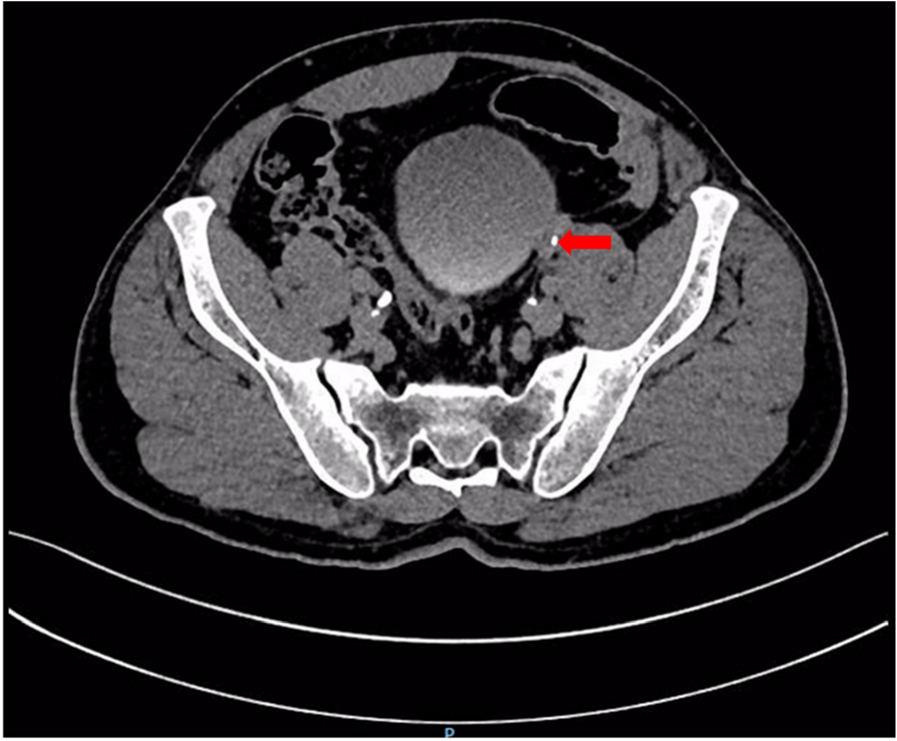
A nodule-shaped high-density shadow in the ileal lumen of the left ureter (indicated by the arrow)

**Fig. 4:**
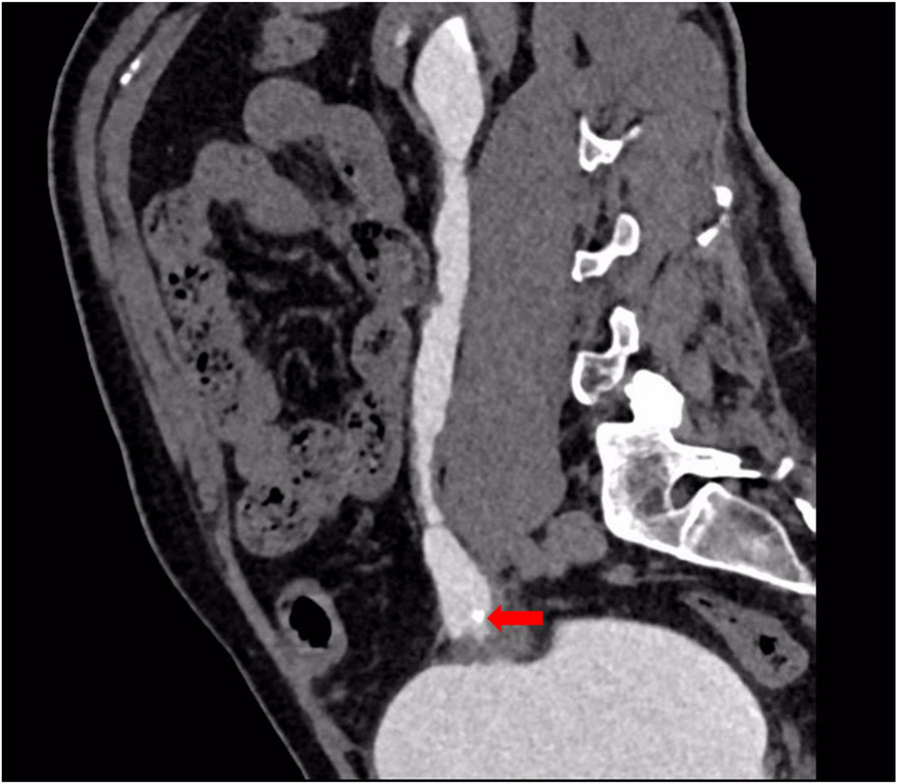
A nodule-shaped high-density shadow in the ileal lumen of the left ureter (indicated by the arrow)

Ten months after the Yang-Monti ileal ureter reconstruction, the patient developed left lumbar pain and discomfort. Noncontrast computed tomography showed that the left kidney had a slightly smaller volume than the right, and that the left renal pelvis and renal calices were slightly expanded and hydronephrotic. Two nodule-shaped high-density shadows were present in the lower renal calyx; the diameter of the larger shadow was 4 to 5 mm. A liquid-density shadow was seen in the ileal lumen of the left ureter, with a small nodule-shaped high-density shadow (3-mm diameter) at its end. Corticomedullary development was good. During excretion, accumulation of contrast agent could be seen in the left renal pelvis, renal calices, and ureter (Figs. 2, 3 and 4). The serum creatinine concentration was normal.

**Fig. 5:**
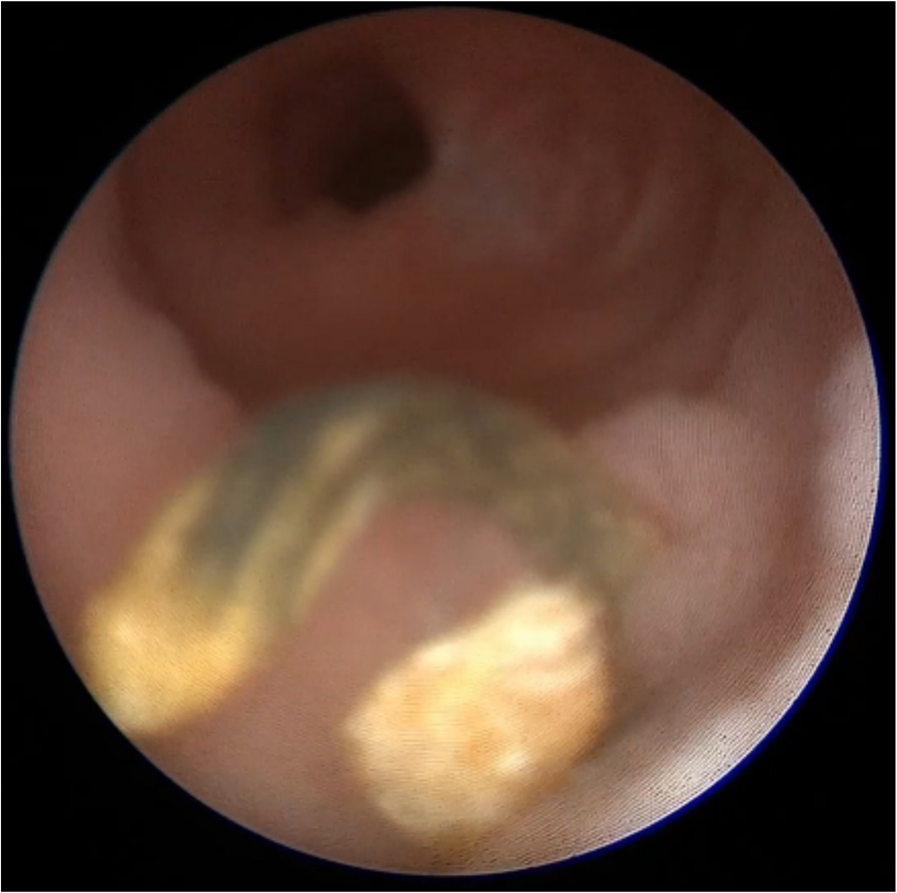
The calculi were attached to surgical sutures, and the sutures were embedded under the ileal ureteral mucosa

**Fig. 6:**
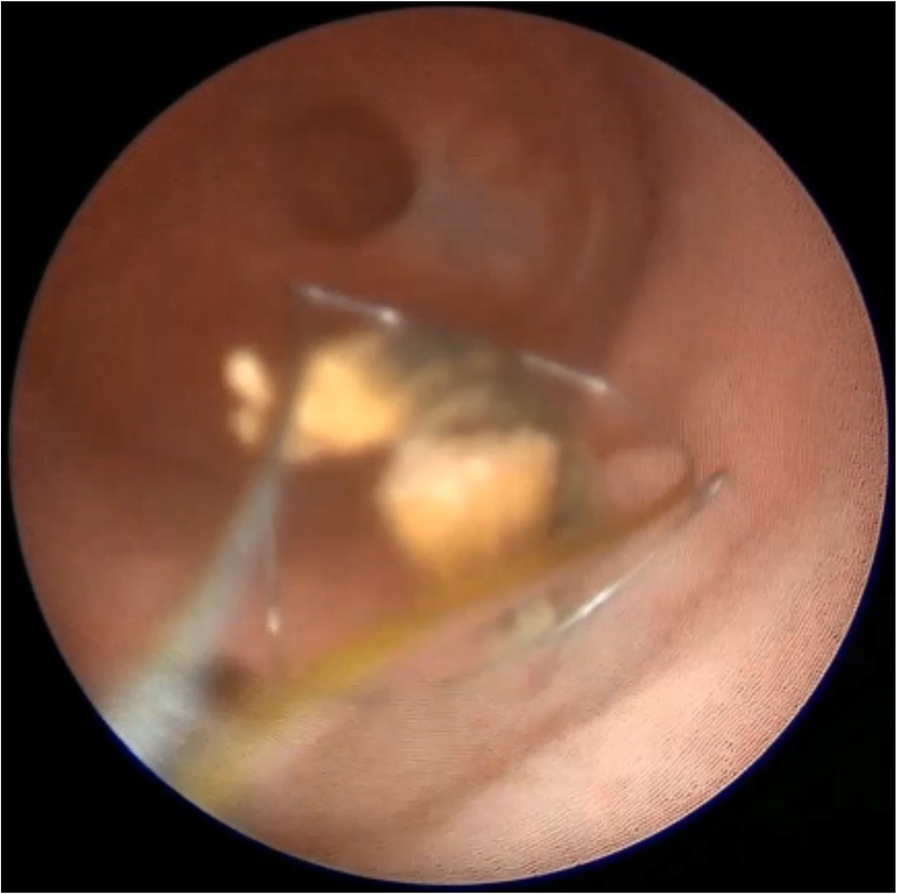
A basket extractor was used to hold the calculi and sutures

**Fig. 7:**
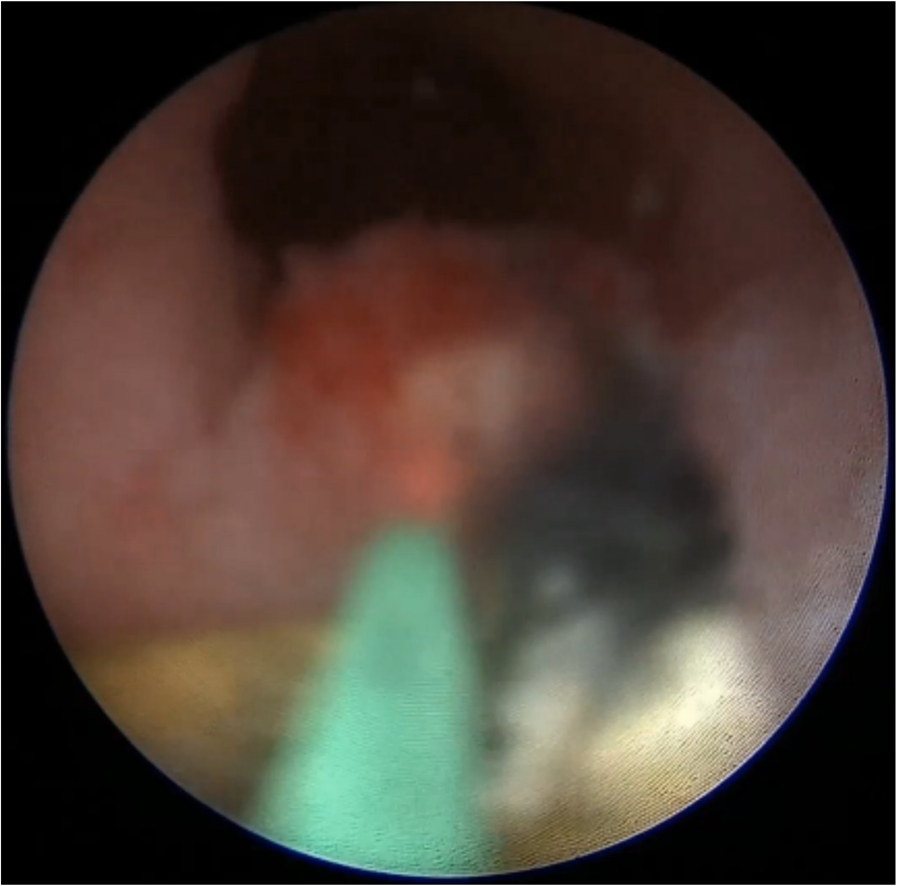
The calculi and sutures were broken up with a holmium laser

**Fig. 8:**
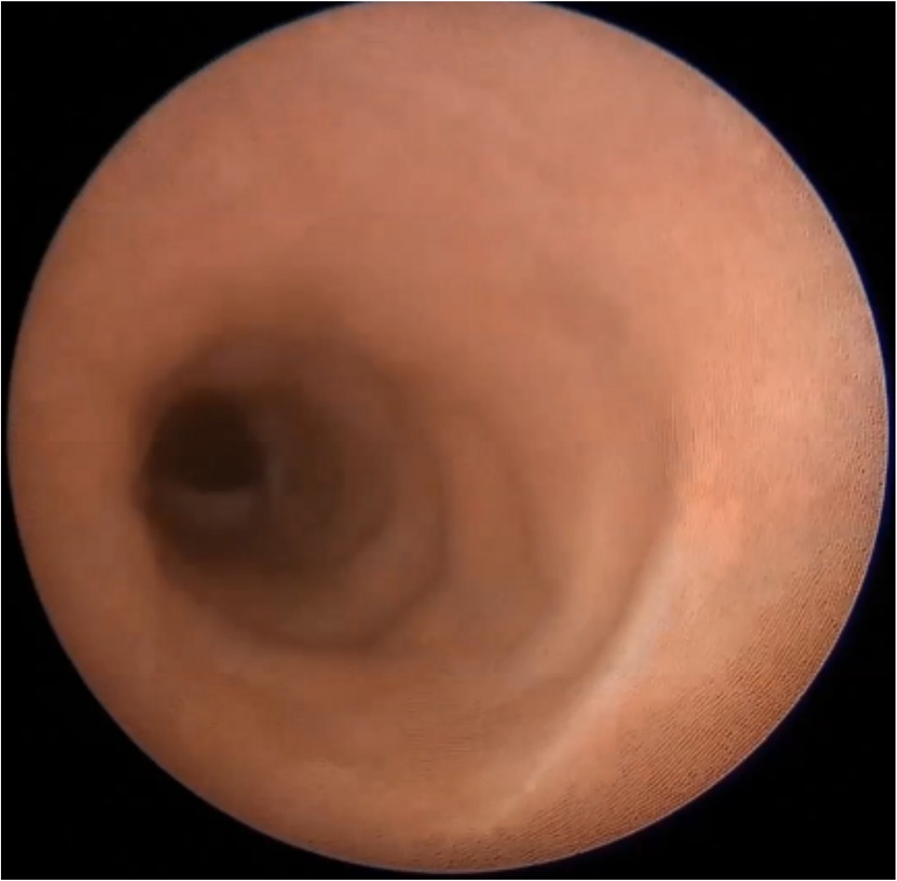
The Yang-Monti ileal ureter had a thick and straight lumen and smooth mucosa

The patient was placed in the lithotomy position under general anesthesia. The ileal ureterovesical reimplantation opening was smoothly entered under direct vision using a 9.5-Fr semirigid ureteroscope (Richard Wolf Medical Instruments, Knittlingen, Germany). Ureteroscopic examination revealed that the calculi were attached to surgical sutures located from 1 cm from the inner end of the ileal ureter to the ureteral opening, and that the sutures were embedded under the ileal ureteral mucosa in a ring shape (Fig. 5). A basket extractor [NGage Nitinol Stone Extractor (NGE-017115), 1.7 Fr × 115 cm; Cook Medical, Bloomington, IN, USA] was used to hold the calculi and sutures, which were then broken up with a holmium laser (0.6 J, 30 Hz, 200-μm fiber; 60-W LISA Sphinx Holmium:Yttrium-Aluminium-Garnet Laser System, LISA Laser Products, Katlenburg-Lindau, Germany) and completely removed (Figs. 6, 7). Ureteroscopy revealed that the Yang-Monti ileal ureter had a thick and straight lumen, smooth mucosa, and no obvious folds or anastomoses between sections (Fig. 8). Using a Zebra guidewire (Boston Scientific, Marlborough, MA, USA) for guidance, a flexible ureteroscope working sheath (12/14 Fr × 115 cm, M006250226; Boston Scientific) was imbedded and inserted into the left renal pelvis under direct vision using a digital flexible ureteroscope (8.5 Fr, 11278VS; Karl Storz, Tuttlingen, Germany). Using a filling pump (26331020–1; Storz Medical, Tägerwilen, Switzerland) with an infusion pressure of 80 mmHg, the two lower calyceal calculi were identified, broken up, and removed. As a preoperative urine culture had revealed a urinary tract infection, antibiotics were administered postoperatively. Additionally, a 5-Fr ureteral catheter (Kangge Medical Instruments, Shanghai, China) and a urinary catheter were left indwelling, and were removed on postoperative day 3.

## Discussion and conclusion

A previous study investigating minimally invasive treatment of ureteral calculi following ileal neobladder surgery reported that the cause of calculi development may be related to the presence of foreign bodies (e.g., ureteral stents and sutures) [1]. Furthermore, the generation of ileal ureteral calculi also has a very close relationship with intestinal mucous [2]; when the ileal anastomosis is bulky, the intestinal mucous secreted in the ileal segment near the kidney during bowel movements is more likely to enter the kidney, accumulate in the renal pelvis within the calyx, and become a matrix or core of stone formation, along with urine salt deposition and stone formation [2]. If the ileal replacement ureter is too long and the postoperative replacement urine becomes stored in the ileum, this urine combines with the large amount of mucus secreted by the intestinal tract to form calculi, and ureteral calculi in the ureteral diversion may be formed by renal calculi falling through the urine drainage [3]. In the present case, the absorbable suture in the lower ileal ureteral segment from the previous ureteral reconstruction had not degraded or fallen off, and so served as a matrix or core for calculi formation; its long-term contact with urine resulted in urinary salt being continuously deposited to form calculi [4, 5]. The lower ileal ureteral calculi were successfully removed using a rigid ureteroscope combined with holmium laser lithotripsy to remove the sutures and attached calculi.

During the holmium laser removal of sutures, it is important to determine the shape and direction of the suture, cut the suture from its root near the ileal ureteral mucosa, and check the integrity of the suture to prevent leaving residual suture that will again enable calculi to form. Suture removal must be performed under direct vision. As the suture moves along with the movement of the perfusate, the suture must be fixed with a basket extractor to create adequate tension and thus prevent the suture from moving; this improves the success rate of suture removal, and reduces the risk of ileal ureteral injury.

A digital flexible ureteroscope was entered into the left renal collection system. As the calculi were small (maximum diameter of 5 mm) and oval-shaped with smooth surfaces, we were able to use a basket extractor to cover and remove the calculi. During removal, we discovered that the calculi had diameters that were larger than that of the working sheath for the flexible ureteroscope; hence, we covered each calculus and removed it together with the working sheath of the flexible ureteroscope. Using intraoperative ureteroscopic guidance, a 5-Fr double-J stent was placed upon completion of the surgery.

The key points gained from our experience are as follows. (1) The thinnest holmium laser fiber with the appropriate energy for cutting should be selected, so that the laser fiber can pass through the gap between the basket extractor and the ureteroscopic operation channel. It is best to conduct an in vitro analysis of suture materials, and then choose the best energy setting for cutting. The appropriate energy setting is that which can cut the suture while preventing damage to the ureter. (2) Good intraoperative control of the perfusion pressure must be maintained. Excessive pressure causes dilatation of the reconstructed ureteral lumen, increased pressure in the renal pelvis and renal calices, and an increased risk of postoperative infection. (3) The ileal ureter has a large lumen diameter. Even if the diameter of the intestinal canal is cut using the Yang-Monti method, the reconstructed ureteral lumen still has a large diameter, and thus the rigid ureteroscope, flexible ureteroscope, and working sheath of the flexible ureteroscope can be successfully retrograded to the renal pelvis. When the diameters of the calculi are smaller than the diameter of the ureteral lumen, a basket extractor can be used.

In summary, ureteroscopy combined with laser lithotripsy seems to be a valuable option for the management of urinary calculi following Yang-Monti ileal ureter reconstruction.
